# Estimation of the Proportion and Determinants of Diabetes Mellitus Among Notified Tuberculosis Patients in Jaipur, Rajasthan, India

**DOI:** 10.7759/cureus.80319

**Published:** 2025-03-10

**Authors:** Janesh Kumar Gautam, Anjali Thanvi, Purushotam Soni, Praveen K Anand

**Affiliations:** 1 Biotechnology, Indian Council of Medical Research-National Institute for Implementation Research on Noncommunicable Diseases, Jodhpur, IND; 2 Public Health, Indian Council of Medical Research-National Institute for Implementation Research on Noncommunicable Diseases, Jodhpur, IND; 3 Public Health, Government of Rajasthan, Jaipur, IND; 4 Epidemiology, Indian Council of Medical Research-National Institute for Implementation Research on Noncommunicable Diseases, Jodhpur, IND

**Keywords:** determinants, diabetes mellitus, india, jaipur, treatment outcomes, tuberculosis, tuberculosis-diabetes mellitus comorbidity

## Abstract

Background: Tuberculosis (TB) and diabetes mellitus (DM) comorbidity is a significant public health problem globally and in India. The present study estimated the proportion and determinants of DM among notified TB patients within the Indian National Tuberculosis Elimination Program (NTEP).

Methods: The present study is the secondary data analysis of the NTEP data obtained from the District Tuberculosis Office, Jaipur, Rajasthan, India. The total number of TB patients included in the study was 4679. Sociodemographic and clinical data were compared between TB-DM and TB-only patients. Bivariate chi-squared analysis and multivariate logistic regression analysis were employed to understand the determinants of TB-DM comorbidity.

Results: The proportion of DM among the notified TB patients was found to be 0.98% (46). TB-DM patients were significantly older (a mean age of 51.6±12.4 years vs. 34.2±17.3 years; p<0.001) and had higher body weight (51.2±11.4 kg vs. 46.2±12.4 kg; p<0.05) than TB-only patients. Bivariate analysis revealed that males and individuals over 35 years of age had higher odds of TB-DM, with odds ratios of 1.906 (95% CI: 1.015-3.582) and 29.871 (95% CI: 7.233-123.363), respectively. Multivariate binary logistic regression analysis determined that age >=35 years was a significant determinant of TB-DM comorbidity (adjusted odds ratio (AOR): 28.641; 95% CI: 6.818-120.313; p<0.001). There was no significant association of treatment success rate and death rate with TB-DM comorbidity. The comparison of diagnostic and enrollment facilities in diagnosing and enrolling TB-DM revealed that the diagnostic and enrollment of TB-DM patients were higher in private healthcare facilities than in public healthcare facilities.

Conclusion: The study results determined that age >35 years is the significant determinant of TB-DM comorbidity. The analysis of diagnostic and enrollment facilities showed that TB-DM patients were more likely to be diagnosed and enrolled in private hospitals than government healthcare facilities. The study findings suggest that there is a need for integrated management approaches that address TB and DM concurrently.

## Introduction

Tuberculosis (TB) is the 10th major cause of death globally [1]. India accounts for 28% of the world's TB incident cases in 2021. In parallel, the global prevalence of diabetes mellitus (DM) is rising at an alarming rate, particularly in developing nations. India, for example, not only bears the highest burden of TB but also has the second-highest number of individuals affected by DM [1,2].

The interaction between TB and DM is increasingly recognized as a critical public health concern. DM predisposes individuals to active TB by compromising the immune response, while TB infection can further destabilize glycemic control in diabetic patients [3,4]. Moreover, DM has been associated with poorer TB treatment outcomes, including higher rates of treatment failure and mortality, likely due to underlying metabolic and immunological disturbances [5,6].

The occurrence of DM among TB patients has been reported from different parts of the world [7,8]. The clinicopathological profile and treatment outcomes differ in the case of TB-DM comorbidity as compared to TB [9]. The prevalence of TB-DM comorbidity has increased in the past two decades in India [10]. Previous studies from India reported that the prevalence of TB-DM comorbidity ranges from 6.3% to 54.1% [10,11]. Recent studies have shown that TB-DM comorbidity is associated with increased treatment failure and higher mortality [12]. A study in Yemen recommended enhanced screening, suitable drugs, and better treatment strategies to reduce the burden of TB-DM comorbidity [13]. 

Within India, TB-DM comorbidity poses challenges for disease control and patient management. Despite robust efforts under the Indian National Tuberculosis Elimination Program (NTEP), the dual burden of TB-DM remains inadequately addressed, with limited data on the sociodemographic and clinical factors influencing this comorbidity. Understanding these factors is crucial for developing integrated strategies to improve screening, diagnosis, and treatment outcomes.

The present study aimed to estimate the proportion and determinants of DM among notified TB patients within the Indian NTEP. The treatment outcomes of TB-DM comorbidity were also analyzed. The study also assesses the differences in diagnostic and enrollment facilities in diagnosing and enrolling TB-DM comorbid patients. The study's results inform public health policy and practice, thereby contributing to the more effective management of TB in the context of the growing DM epidemic.

## Materials and methods

Study design and settings

A retrospective study was conducted using data collected under the NTEP. The study data was obtained from the District Tuberculosis Office, Jaipur, functioning under NTEP. The present study is the secondary data analysis of the NTEP data obtained from the District Tuberculosis Office, Jaipur, Rajasthan, India. The data collected for around one-year duration (January 2021 to March 2022) was used in the study.

Study population

A total of 4679 TB patients were included in the analysis. The inclusion criteria consisted of TB patients who underwent DM screening, while exclusion criteria included incomplete records or missing key demographic or clinical data. DM was diagnosed using blood sugar levels, while TB was confirmed through the Cartridge-Based Nucleic Acid Amplification Test (CBNAAT) testing.

Data variables

Sociodemographic variables (age, weight, gender, and residence area) were analyzed to understand the association with TB-DM. The age categorization was based on the study population's characteristics. The mean age of notified TB patients in our study was 34.2 years, which guided our choice of this cutoff. The treatment success rate and death rate were analyzed to determine their association with TB-DM. The analysis of the comparison of diagnostic and enrollment facilities in diagnosing and enrolling TB-DM was done in the study.

Statistical analysis

The data was analyzed using IBM SPSS Statistics for Windows, V. 28.0 (IBM Corp., Armonk, NY, USA). The chi-squared test was employed for bivariate analysis, whereas multivariate analysis was performed using binary logistic regression. The adjusted odds ratio (AOR) or odds ratio (OR) with a 95% confidence interval (CI) was calculated. Student's t-tests were employed to compare the means between the two quantitative variables. P-values <0.001 and <0.05 were considered to be statistically significant.

Ethical consideration

The present study is a secondary data analysis. All patient data were anonymized to ensure confidentiality.

## Results

Sociodemographic characteristics of the study participants

Table 1 represents the sociodemographic characteristics of the study participants, which are all TB patients included in the study. Out of 4679 total TB patients, the majority (2559 (54.7%)) were males. The majority (2346 (50.1%)) of TB patients belong to the age group of 15-34 years. Most of the TB patients (2489 (53.2%)) included in the study resided in the urban areas of Jaipur, Rajasthan, India.

**Table 1:** Sociodemographic characteristics of the study participants

Variables	Total (n=4679)
Gender	
Male	2559 (54.7%)
Female	2120 (45.3%)
Age group (in years)	
0-14	324 (6.9%)
15-34	2346 (50.1%)
35-59	1412 (30.2%)
>60	597 (12.8%)
Residence area	
Rural	2156 (46.1%)
Urban	2489 (53.2%)
Missing values	34 (0.7%)

Proportion of DM among the notified TB patients

Table 2 shows that out of 4679 total patients with TB, only 46 (0.98%) had DM-TB comorbidity. The proportion of DM among the notified TB patients was 0.98%. The TB-DM comorbidity was higher among males (1.3%) compared to females (0.7%). The majority of TB-DM patients were aged above 35 years (2.2%) compared to those aged 0-34 years (0.1%). There was no difference in the TB-DM comorbidity among patients residing in rural vs. urban areas. 

**Table 2:** Proportion of DM among the notified TB patients DM: diabetes mellitus; TB: tuberculosis

Variables	TB-DM (n=46 (0.98%))
Gender	
Male	32 (1.3%)
Female	14 (0.7%)
Age group (in years)	
0-34	2 (0.1%)
≥35	44 (2.2%)
Residence area	
Rural	22 (1%)
Urban	24 (1%)

Comparison of mean age and weight between TB-DM comorbidity and TB

Table 3 shows the comparison of mean age and weight between TB-DM comorbidity and TB. The mean age of the study participants was 34.4±17.40 years, and the mean weight was 46.32±12.44. The mean age of TB-DM patients was 51.6±12.4 years, significantly higher than TB-only patients (34.2±17.3 years; p<0.001). The mean weight of TB-DM patients was also significantly higher (51.2±11.4 kg) compared to TB-only patients (46.2±12.4 kg; p<0.05).

**Table 3:** Comparison of mean age and weight between TB-DM comorbid and TB patients Independent samples t-test was employed to compare the mean age and weight between TB-DM comorbid and TB patients. SD: standard deviation; DM: diabetes mellitus; TB: tuberculosis

	Total (n=4679)	TB-DM (n=46)	TB (n=4633)	Statistical test	P-value
Age (years), mean±SD	34.4±17.40	51.6±12.4	34.2±17.3	Independent samples t-test	<0.001
Weight (kg), mean±SD	46.32±12.44	51.2±11.4	46.2±12.4	Independent samples t-test	<0.05

Association of TB-DM comorbidity with sociodemographic factors

Table 4 shows the association of TB-DM comorbidity with sociodemographics through bivariate chi-squared analysis. 1.3% (32) of males had TB-DM as compared to 0.7% (14) of females (p<0.05). Males have significantly higher odds of TB-DM with an odds ratio of 1.906 (1.015-3.582) compared to females. The highest proportion of TB-DM was observed in patients older than 35 years (2.2%), compared to 0.1% in those aged 0-34 years (p<0.001). Patients belonging to the age group of >=35 years have significantly higher odds of TB-DM, with an odds ratio of 29.871 (7.233-123.363). There was a non-significant association of residence with TB-DM comorbidity. 

**Table 4:** Association of TB-DM comorbidity with sociodemographic factors through bivariate analysis Bivariate chi-squared analysis determined the association of gender, age group, and residence area on TB-DM comorbidity. OR: odds ratio; CI: confidence interval; χ²: Pearson's chi-squared test; DM: diabetes mellitus; TB: tuberculosis

		TB-DM (n=46)	TB (n=4633)	Chi-squared (χ²) value	P-value	OR (95% CI)	Risk ratio (95% CI)
Gender	Female (n=2120)	14 (0.7%)	2106 (99.3%)	4.16	<0.05	1.906 (1.015-3.582)	1.006 (1.000-1.012)
	Male (n=2559)	32 (1.3%)	2527 (98.7%)				
Age group (in years)	0-34 (n=2670)	2 (0.1%)	2668 (99.9%)	52.69	<0.001	29.871 (7.233-123.363)	1.022 (1.015-1.028)
	>=35 (n=2009)	44 (2.2%)	1965 (97.8%)				
Residence area	Rural (n=2156)	22 (1%)	2134 (99%)	0.037	0.85	-	-
	Urban (n=2489)	24 (1%)	2465 (99%)				

Table 5 shows the association of TB-DM comorbidity with sociodemographics through multivariate binary logistic regression. Multivariate logistic regression identified age >=35 years as a significant determinant of TB-DM (AOR: 28.641; 95% CI: 6.818-120.313; p<0.001). Gender, residence, and weight were not significantly associated with TB-DM.

**Table 5:** Association of TB-DM comorbidity with sociodemographic factors through multivariate regression analysis Binary logistic regression was employed to understand the determinants of TB-DM comorbidity. AOR: adjusted odds ratio; CI: confidence interval; DM: diabetes mellitus; TB: tuberculosis

	P-value	AOR	95% CI
Age (reference: 0-34)	<0.001	28.641	6.818-120.313
Weight	0.296	1.011	0.990-1.032
Male (reference: female)	0.976	1.010	0.530-1.925
Residence (reference: rural)	0.548	1.198	0.665-2.159

Treatment outcomes in TB-DM and TB patients

Table 6 represents the comparison of treatment outcomes in TB-DM and TB patients. Bivariate chi-squared analysis was employed to understand the association of treatment success rate and death rate with TB-DM comorbidity. There was no significant association of treatment success rate and death rate with TB-DM comorbidity.

**Table 6:** Comparison of the treatment outcome between TB-DM comorbidity and TB Bivariate chi-squared analysis was employed to understand the association of treatment success rate and death rate with TB-DM comorbidity. χ²: Pearson's chi-squared test; DM: diabetes mellitus; TB: tuberculosis

		TB-DM (n=46)	TB (n=4633)	Total (n=4679)	Chi-squared (χ²) value	P-value
Treatment success rate	Treatment success	40 (1%)	4155 (99%)	4195	0.37	0.54
	Treatment failure	6 (1.2%)	478 (98.8%)	484		
Death rate	Died	3 (1.4%)	216 (98.6%)	219	0.35	0.55
	Not died	43 (1%)	4417 (99%)	4460		

Comparison of diagnostic and enrollment facility in diagnosing and enrolling TB-DM

Table 7 represents the diagnostic and enrollment facility analysis. Out of all the TB patients diagnosed in private healthcare facilities (n=210), 3.3% were TB-DM, and 96.7% were TB-only. In the case of government healthcare facilities, out of all TB patients diagnosed (n=4469), 0.9% were TB-DM and 99.1% were TB-only. TB-DM patients were more likely to be diagnosed in private healthcare settings (3.3%) compared to TB-only patients (0.9%) (p<0.001). Out of all the TB patients enrolled in the private healthcare facilities (n=158), 2.5% were TB-DM, and 97.5% were TB-only. In the case of government healthcare facilities, out of all the TB patients diagnosed (n=4521), 0.9% were TB-DM, and 99.1% were TB-only. Enrollment in private facilities was also higher among TB-DM patients (2.5%) compared to TB-only patients (0.9%) (p<0.05). No significant differences were observed in diagnosis or enrollment based on rural versus urban residence.

**Table 7:** Comparison of diagnostic and enrollment facilities in diagnosing and enrolling TB-DM comorbid patients Bivariate chi-squared analysis was employed to compare the diagnostic and enrollment facilities in diagnosing and enrolling TB-DM comorbid patients. DM: diabetes mellitus; TB: tuberculosis

	TB-DM (n=46)	TB (n=4633)	Total (n=4679)	Chi-squared (χ²) value	P-value
Diagnostic facility					
Rural area	10 (1.5%)	663 (98.5%)	673	2.04	0.15
Urban area	36 (0.9%)	3970 (99.1%)	4006		
Diagnostic facility type					
Government	39 (0.9%)	4430 (99.1%)	4469	12.48	<0.001
Private	7 (3.3%)	203 (96.7%)	210		
Enrollment facility					
Rural area	14 (1.3%)	1052 (98.7%)	1066	2.66	0.26
Urban area	32 (0.9%)	3348 (99.1%)	3480		
Enrollment facility type					
Government	42 (0.9%)	4479 (99.1%)	4521	4.03	<0.05
Private	4 (2.5%)	154 (97.5%)	158		

## Discussion

The TB-DM comorbidity has been reported in many parts of the world, including India [7,8,14]. A previous study recommended the dual diagnostics of TB and DM and optimal care of TB-DM comorbid patients for reducing the burden of TB-DM comorbidity [15]. Previous studies have reported that the prevalence of TB-DM comorbidity varied from 6.3% to 54.1% [10,11,16]. NTEP India TB Report 2024 reported that the prevalence of TB-DM comorbidity was 7.7% in India [17]. While TB-DM comorbidity is a recognized issue, TB-DM notified proportion and associated determinants had not been previously studied in Jaipur, Rajasthan, making this analysis relevant to local clinical practice.

Previous studies on the knowledge of TB services and active screening of TB have been done in Jaipur, Rajasthan [18,19]. The NTEP India TB Report 2022 reported that the prevalence of TB-DM comorbidity was 2.6% in Rajasthan [17]. Regarding the DM burden in Jaipur, previous studies have reported a prevalence of 17.50% in the region [20]. However, data on the comorbidity of TB-DM is in its first steps in Jaipur, Rajasthan. Our study investigated the prevalence, determinants, and treatment outcomes of TB-DM comorbidity among patients enrolled under the NTEP.

A key finding of our study is that TB-DM patients were significantly older and had higher body weights compared to TB-only patients. The robust association between age >35 years and TB-DM (AOR: 29.87; p<0.001) aligns with previous reports indicating that the metabolic derangements associated with aging predispose individuals to DM, which in turn increases the risk for developing active TB [3,4]. The bivariate analysis also revealed that males had a higher proportion of TB-DM comorbidity. However, multivariate analysis confirmed that higher age is the major determinant of TB-DM comorbidity. Although not statistically significant, the mortality rate was higher in TB-DM patients, and the overall treatment success was significantly lower in TB-DM comorbidity. This suggests that DM may adversely impact the management of TB, potentially due to complications related to metabolic misregulation, immunity, or drug interactions. Thus, there is a need for integrated management approaches that address TB and DM concurrently.

The analysis of diagnostic and enrollment facilities showed that TB-DM patients were more likely to be diagnosed and enrolled in private hospitals compared to government healthcare facilities. The diagnostic and enrollment facilities of DM among the TB patients are fully sponsored by the Indian NTEP at private and public healthcare facilities, ensuring no financial burden on the patients [21]. The higher utilization of private healthcare facilities by TB-DM comorbid patients could be attributed to the perception of a better standard of care in the private sector. The differences in healthcare-seeking patterns and access to diagnostic services could be a plausible explanation for this study result. Additionally, social status may play a role, as individuals with higher socioeconomic status may prefer private healthcare facilities. The relapse rate of TB among TB-DM comorbid patients was previously reported to be higher [22]. The study recommends that to mitigate the relapse rate, TB patients aged 35 years and above should be subjected to sensitive DM diagnosis tests like the oral glucose tolerance test and hemoglobin A1C (HbA1c) test for better TB-DM diagnosis and close monitoring of the treatment.

Strengths and limitations of the study

The data on the comorbidity of TB-DM is in its first steps in Jaipur, Rajasthan. The present study analyzed the large NTEP dataset (n=4679) to estimate the proportion and determinants of DM among the notified TB patients in Jaipur, Rajasthan. The associated determinants were estimated through both bivariate chi-squared and multivariate regression analyses. The limitation of the study is the retrospective nature of the study using NTEP data, which may be susceptible to under-reporting or misclassification bias. The low proportion of TB-DM comorbid patients (0.98%) may limit the statistical analysis.

## Conclusions

The proportion of DM among the notified TB patients was found to be 0.98%. TB-DM patients were significantly older and had higher body weight than TB-only patients. Bivariate analysis revealed that males and individuals over 35 years of age had higher odds of TB-DM. Multivariate binary logistic regression analysis determined that age ≥35 was a significant and independent determinant of TB-DM comorbidity. The analysis of diagnostic and enrollment facilities showed that TB-DM patients were more likely to be diagnosed and enrolled in private hospitals than government healthcare facilities. The study findings suggest that there is a need for integrated management approaches that address TB and DM concurrently.
